# Total Antioxidant Status in Type 2 Diabetic Patients in Palestine

**DOI:** 10.1155/2015/461271

**Published:** 2015-05-27

**Authors:** Akram T. Kharroubi, Hisham M. Darwish, Mutaz A. Akkawi, Abdelkareem A. Ashareef, Zaher A. Almasri, Khaldoun A. Bader, Umaiyeh M. Khammash

**Affiliations:** ^1^Department of Medical Laboratory Sciences, Faculty of Health Professions, Al-Quds University, Jerusalem, State of Palestine; ^2^Department of Biochemistry, Faculty of Medicine, Al-Quds University, Jerusalem, State of Palestine; ^3^Department of Biological Sciences, Faculty of Science and Technology, Al-Quds University, Jerusalem, State of Palestine; ^4^Faculty of Public Health, Al-Quds University, Jerusalem, State of Palestine; ^5^United Nation Relief and Works Agency (UNRWA), Jerusalem, State of Palestine

## Abstract

The objective of this study was to compare the level of total antioxidant status (TAS) in type 2 diabetic and normal Palestinian subjects as well as the major factors influencing TAS levels. A sample of convenience composed of 212 type 2 diabetic and 208 normal subjects above the age of 40 were recruited. Only 9.8% of the subjects had normal body mass index (BMI) levels (<25), 29% were overweight (≥25 to <30), and 61.2% were obese (≥30). The mean levels of TAS were significantly higher in diabetic compared to control subjects (2.18 versus 1.84 mM Trolox, *P* = 0.001) and in hypertensive subjects compared to subjects with normal blood pressure (BP). Mean TAS levels were higher in obese compared to nonobese subjects (2.12 versus 1.85 mM Trolox, *P* = 0.001). Mean TAS levels were similarly higher in subjects with high fasting plasma glucose (FPG) compared to normal FPG (2.19 versus 1.90 mM Trolox) and high HbA1c (≥6.5%) compared to HbA1c < 6.5% (2.14 versus 1.91 mM Trolox). Multivariate analysis revealed that only diabetic status (*P* = 0.032) and the level of education (*P* = 0.036) were significantly associated with TAS. In conclusion diabetic patients had 18.5% increase in TAS levels compared to control subjects.

## 1. Introduction

The prevalence of diabetes in the Middle East countries is among the highest in the world [1, 2]. The prevalence of diabetes among the Palestinian population is about 12% [3, 4] compared with the highest (about 20%) in the United Arab Emirates [5, 6]. Four of the top ten countries with the highest prevalence of prediabetes are in the Middle East Arab states of the Gulf (Kuwait, Qatar, United Arab Emirates (UAE), and Bahrin with prevalence of 17.9%, 17.1%, 16.6%, and 16.3%, resp.) [2]. Risk factors for type 2 diabetes mellitus among the Palestinian community including obesity, genetic predisposition, and sedentary life style are clearly evident [7]. Oxidative stress, defined as excess formation and/or insufficient removal of highly reactive molecules such as reactive oxygen species (ROS) and reactive nitrogen species (RNS), increases in diabetes when free radical production exceeds the body's ability to neutralize them [8, 9]. Excess generation of free radicals has been associated with tissue damage and complications in diabetic patients [10–19].

Despite the agreement on the increase of free radicals in diabetic patients, the level of antioxidants in diabetic patients has been reported to decrease [20–25], increase [26–28], or stay the same [10]. The effect of diabetes on total antioxidant levels seems to be complicated by the effect of diabetes on individual antioxidant systems. Kimura et al. [27] reported an increase in extracellular superoxide dismutase (SOD) whereas Palanduz et al. [29] reported a decrease in plasma glutathione peroxidase levels and an increase in plasma SOD at the same time. Al-Rawi [30] also reported an increase in SOD in diabetic patients in the UAE.

In this study we investigated for the first time the association of TAS in Palestinian type 2 diabetic subjects with several environmental and biochemical parameters known to affect or be affected by diabetes.

## 2. Materials and Methods

### 2.1. Participants

A sample of convenience composed of 209 known type 2 diabetic patients treated at UNRWA clinics for diabetes (70 males and 139 females) and 208 known normal subjects (68 males and 140 females) above the age of 40 were recruited from three major central clinics in the West Bank administered by UNRWA. Seventy-six out of the 208 normal subjects have impaired fasting plasma glucose (≥5.6 to <7.0 mmol/L). All subjects were instructed to fast for 10–12 hours before coming to the clinics at 8:00 am. A special questionnaire designed to collect sociodemographic, lifestyle, family history, and health related information was filled for all participants during direct interviews with the researchers. Subjects who administered antioxidants as supplements like vitamins were excluded from the study. Diabetic subjects were on different medications, mainly metformin. Those who were on insulin were excluded from the study. Blood pressure (BP), weight, and height were measured by the medical teams in the clinics. Body mass index (BMI) in kg/m^2^ was categorized as normal (BMI < 25), overweight (BMI ≥ 25 to <30), and obese (BMI ≥ 30). Diabetic subjects were not on special diets. The study protocol was approved by Al-Quds University and UNRWA ethical committees and all participants gave their informed consent.

### 2.2. Analytical Procedures

Blood samples were collected from all subjects and were tested for their total antioxidant status (TAS), fasting plasma glucose (FPG), glycated hemoglobin (HbA1c), and total lipid profile including total cholesterol (TC), triacylglycerol (TG), low density lipoprotein-cholesterol (LDL-C), and high density lipoprotein-cholesterol (HDL-C). Serum for TAS analysis was separated using refrigerated centrifuge under dim light and kept frozen at −80°C for two weeks before analysis.

The antioxidant activity was measured as the ability of the serum to prevent ABTS oxidation in comparison to Trolox and quantified as millimolar Trolox equivalents using antioxidant assay kits (Cayman Chemical Co., Ann Arbor, MI, USA). Glycated hemoglobin was measured by boronate affinity assay using NycoCard HbA1c Kit (Axis-shield, Oslo, Norway) that reports a standardized HbA1c value according to IFCC recommendations. Fasting plasma glucose and total lipid profile (TC, TG, and HDL) were measured enzymatically using Chemwell chemistry analyzer (Awareness Tech, USA), and LDL-C was calculated from the equation of Friedewald (LDL-C = TC − [HDL-C + (TG/5)]). The FPG results were categorized as normal (FPG < 100 mg/dL), impaired (FPG 100–125 mg/dL), and diabetic (FPG ≥ 126 mg/dL).

### 2.3. Statistical Analysis

Statistical analysis was performed using SPSS 17.0 software (SPSS Inc., Chicago, IL, USA). Pearson's correlation coefficient was used to test for colinearity between the continuous variables; statistical comparisons between different groups for these continuous variables were carried out using Student's *t*-test and ANOVA and Pearson's chi-square statistic was used to assess relationships between categorical variables. Multivariate analysis implementing general linear model (ANCOVA) was performed to adjust and control potential confounders and to test for interactions between the variables that appeared significantly associated with the independent variable (TAS) in the bivariate analysis. Statistical significance was accepted at *P* < 0.05. Because of missing values the number of each group in different comparisons is different.

## 3. Results

Table 1 shows that mean FPG and HbA1c in diabetic subjects were higher than controls (10.3 versus 5.31 mmol/L for FPG and 8.14 versus 5.75% for HbA1c, resp.). Evidently, the mean systolic and diastolic BP were significantly higher in diabetic compared to normal subjects (Table 1). In addition, the mean triacylglycerol levels were higher whereas HDL levels were significantly lower in diabetics compared to normal subjects. Table 1 also shows that BMI for both diabetic and control subjects was above 30 which indicates that control and diabetic subjects were both obese. Diabetic subjects had significantly higher mean BMI compared to control subjects (33.3 versus 31.1, resp.). Only 9.8% of the subjects had normal BMI levels whereas 29% were overweight and 61.2% were obese. In our study, subjects with high systolic BP (≥140 mm Hg) constitute 31.6% whereas those with high diastolic BP (≥90 mm Hg) constitute 24.8%. Furthermore, subjects with abnormal lipid profile including high TC (≥5.5 mmol/L), high TG (≥2.0 mmol/L), and high LDL (≥3.5 mmol/L) represented 35.0%, 31.9%, and 33.6%, respectively, whereas subjects with low HDL (<1.0 mmol/L) represented 26.6% of the entire subjects in the study. Moreover, subjects with family history for diabetes or family history for cardiovascular disease (CVD) were 59% and 46% of all the studied subjects, respectively. Our data showed that 64% of subjects with family history for diabetes also developed diabetes compared to 31% for those subjects with no such family history for diabetes (*P* = 0.001). However, the percentage of subjects who developed diabetes with family history for CVD did not significantly differ between diabetic and control subjects (51% versus 48%, *P* = 0.298). As expected, obesity was positively associated with the diabetes where 58% of obese subjects had diabetes compared to 38% of nonobese individuals (*P* = 0.001).

Table 2 shows that the mean levels of TAS are significantly higher in diabetic subjects compared to control subjects (2.18 versus 1.84 mM Trolox resp., *P* = 0.001). Mean TAS levels were also significantly higher in hypertensive subjects (systolic BP ≥ 140 mm Hg or diastolic BP ≥ 90) compared to subjects with normal BP (systolic < 140 mm Hg or diastolic BP < 90 mm Hg). In addition obese subjects had higher mean TAS levels compared to nonobese subjects (normal and overweight) [2.12 ± 0.84 versus 1.85 ± 0.70 mM Trolox resp., *P* = 0.001]. Mean TAS levels were also similarly higher in subjects with high FPG (FPG ≥ 7.0 mmol/L) and HbA1c (HbA1c ≥ 6.5). Evidently, subjects with family history for diabetes or family history for CVD had significantly higher mean levels of TAS compared to subjects without family history for diabetes or CVD. In our study, the abnormal lipid profile among diabetic subjects does not seem to affect TAS levels since subjects with abnormal lipid profile had no significant difference in TAS levels compared to subjects with normal lipid profile (Table 2). Our diabetic subjects had higher mean age compared to normal subjects (55 versus 50, *P* = 0.001). However, when a subset group of 148 subjects with similar age were taken (51.5 ± 5.43 years for diabetic subjects and 51.8 ± 9.13 years for control subjects), the mean levels of TAS in diabetic subjects maintained a significantly higher value compared to control subjects (2.19 ± 0.85 versus 1.86 ± 0.65, *P* = 0.001, resp.). In addition, diabetic subjects had significantly higher mean TAS levels than controls within the obese and overweight groups (2.24 versus 1.95 mM Trolox within the obese and 1.99 versus 1.59 mM Trolox within the overweight subjects, resp.). The educational level seems to significantly affect TAS levels since subjects with higher educational levels (diploma and above) had lower mean TAS levels compared to subjects with lower educational level (less than high school and including illiterates) (1.72 versus 2.09, *P* = 0.007, resp.) This is consistent with the association of educational level with developing diabetes, where only 28% of subjects with higher education level (diploma and above) had diabetes compared to 64% for the illiterate (*P* = 0.001).

Using Pearson's correlation coefficients, Table 3 shows significant relationships between TAS levels and the indicated parameters related to diabetes including FPG (*r* = 0.129, *P* = 0.012), HbA1c (*r* = 0.156, *P* = 0.002,), BMI (*r* = 0.117, *P* = 0.022), and systolic BP (*r* = 0.1, *P* = 0.019). However, when multivariate analysis was performed between TAS as an independent variable and all parameters that were significantly associated with it in the univariate analysis (Table 4), the results revealed that the only parameters that remained to have significant association with TAS were diabetic status (*P* = 0.032) and level of education (*P* = 0.036).

## 4. Conclusions

The implications of increased free radicals and oxidative stress in the development, pathogenesis, and complications of diabetes mellitus [11, 12, 31, 32] and CVD [33] are very strong and well documented despite the inconsistency of the clinical trials using antioxidants in the treatment regimens of diabetes [33–38]. In this study several variables were detected to have an association with the TAS level at the univariate level. Diabetic subjects had 18.5% increase in TAS levels compared to controls.

The effect of diabetes duration on antioxidant levels represents another subject of controversy. In our study, at the univariate level of analysis there was a slight negative correlation between TAS levels and years of diabetes (*r* = −140); however this decrease is not statistically significant. Whiting et al. [39] reported a decrease in the levels of antioxidants in diabetic subjects after 4–6 years of illness while no effect was observed in two years or less. Whether complications of the disease are correlated with the levels of antioxidants is not clear. Opara et al. [22] reported a decrease in antioxidant levels in diabetic subjects with complications while Srivatsan et al. [28] found an increase in antioxidant levels in diabetic subjects without complications. Most diabetic subjects in our study seem to have no obvious complications which is consistent with the notion that the increase in free radicals seems to be associated first with an increase in antioxidant levels and with the progression of the disease the antioxidant levels decrease and complications eventually develop.

The observed significant correlation between TAS with several continuous variables, like systolic BP, FPG, HbA1c, and BMI (*r* = 0.120, 0.129, 0.156, and 0.117, resp.), and noncontinuous variables including education, family history of diabetes, and CVD is relatively not very strong and seems to be mediated through diabetes. This is evident by the multivariate analysis which revealed only diabetes and education to be statistically significant while the effect of education on TAS may also be through diabetes. The multivariate model with these two variables detected as the significant determinants of TAS could have explained about 10% of the variability in TAS levels. This suggests that other determinants might be involved including genetic and environmental factors. It is not clear in our study whether the effect of diabetes on TAS is influenced by the nutritional habits of participating subjects, since fresh vegetables and fruits are major components of the Palestinian food and food is known to affect antioxidant levels [20, 40–43]. The effect of nutritional status on TAS in the Palestinian society needs to be independently evaluated in future studies. One apparent complication in the interpretation of the obtained results in this study is the fact that more than 90% of the participating subjects are either overweight or obese. However, when TAS levels were normalized for BMI, the effect of diabetes on TAS still showed diabetic subjects to have higher levels of TAS which suggest that obesity does not seem to be a primary determinant of the antioxidant status of individuals. It is unlikely that the effect of diabetes on TAS levels is due to medications even though this could not be excluded from this study. Changes of dietary habits of diabetic subjects if present could also affect their TAS levels. It is obvious however that regardless of the effect of diabetes on the levels of antioxidant status, the increase in antioxidant levels in diabetic subjects seems to initially reflect adaptation to high free radicals and the decrease in antioxidant levels apparently reflects high and overwhelming levels of free radicals which eventually may play an important role in the development of diabetes complications. Therefore, under all circumstances, the health benefits of reducing the levels of free radicals by changing dietary habits and supplementation of antioxidant like vitamins in diabetic and nondiabetic subjects should receive more careful evaluation.

## Figures and Tables

**Table 1:** Comparison between diabetic and control subjects with regard to different parameters.

Parameter	Mean ± STD	Mean ± STD	*P*
	Control	Diabetic	
Age (years)	50.0 ± 9.40	55.0 ± 8.31	0.001
Systolic BP (mm Hg)	125 ± 16.8	135 ± 16.9	0.001
Diastolic BP (mm Hg)	77.5 ± 13.8	80.4 ± 12.3	0.021
FPG mmol/L	5.31 ± 0.83	10.3 ± 4.26	0.001
HbA1c (%)	5.75 ± 0.62	8.14 ± 1.74	0.001
BMI	31.1 ± 6.92	33.3 ± 6.18	0.001
TC mmol/L	5.12 ± 1.04	5.30 ± 1.07	0.089
TG mmol/L	1.58 ± 1.34	2.20 ± 1.34	0.001
HDL mmol/L	1.25 ± 0.37	1.09 ± 0.23	0.001
LDL calculated mmol/L	3.19 ± 0.85	3.24 ± 0.98	0.68
LDL direct mmol/L	3.03 ± 1.05	3.26 ± 1.10	0.046

FPG: fasting plasma glucose; TC: total cholesterol; TG: triglycerides; HDL: high density lipoprotein; LDL: low density lipoprotein, BMI: body mass index; HbA1c: hemoglobin A1c.

**Table 2:** Comparison between mean values of total antioxidants status (TAS) with respect to different parameters.

Parameter		TAS (mM Trolox) Mean ± STD (*N*)	*P*
Gender	Male	2.00 ± 0.79 (128)	0.660
	Female	2.04 ± 0.80 (253)	

Diabetic status	Diabetic	2.18 ± 0.86 (206)	0.000
	Control	1.84 ± 0.67 (175)	

Systolic blood pressure (mm Hg)	<140 mm Hg	1.97 ± 0.78 (261)	0.047
	≥140 mm Hg	2.15 ± 0.83 (118)	

Diastolic blood pressure (mm Hg)	<90 mm Hg	1.96 ± 0.79 (285)	0.006
	≥90 mm Hg	2.22 ± 0.81 (94)	

Total cholesterol (mmol/L)	<5.5 mmol/L	2.01 ± 0.77 (254)	0.745
	≥5.5 mmol/L	2.04 ± 0.84 (127)	

HDL (mmol/L)	<1.0 mmol/L	2.02 ± 0.78 (107)	0.959
	≥1.0 mmol/L	2.02 ± 0.80 (274)	

LDL calculated (mmol/L)	<3.5 mmol/L	2.02 ± 0.80 (260)	0.878
	≥3.5 mmol/L	2.03 ± 0.80 (121)	

LDL direct (mmol/L)	<3.5 mmol/L	2.01 ± 0.80 (252)	0.752
	≥3.5 mmol/L	2.04 ± 0.79 (129)	

TG (mmol/L)	<2.0 mmol/L	2.02 ± 0.78 (261)	0.835
	≥2.0 mmol/L	2.02 ± 0.78 (120)	

BMI (kg/m^2^)	<25 (normal)	2.15 ± 0.59 (33)^*∗*^	0.000
	≥25 to <30 (overweight)	1.78 ± 0.70 (113)^*∗∗*^	
	>30 (obese)	2.12 ± 0.84 (235)^*∗*^	

HbA1c (%)	<6.5%	1.91 ± 0.71 (194)	0.005
	≥6.5%	2.14 ± 0.87 (180)	

FPG (mmol/L)	<7.0 mmol/L	1.90 ± 0.74 (220)	0.000
	≥7.0 mmol/L	2.19 ± 0.85 (161)	

Smoking	Current smoker	1.99 ± 0.82 (58)	0.782
	Former smoker	1.96 ± 0.87 (35)	
	Never smoker	2.04 ± 0.79 (281)	

FPG (mmol/L)	Normal	1.80 ± 0.75 (106)^*∗*^	0.000
	Impaired FPG	1.92 ± 0.55 (70)^*∗*^	
	Diabetic	2.17 ± 0.87 (205)^*∗∗*^	

Educational status	Illiterate	2.09 ± 0.76 (69)^*∗*^	0.007
	Less than high school	2.11 ± 0.83 (199)^*∗*^	
	High school	1.96 ± 0.71 (51)	
	Diploma and above	1.72 ± 0.73 (62)^*∗∗*^	

Family history for diabetes	Present	2.08 ± 0.84 (227)	0.049
	Absent	1.92 ± 0.71 (151)	

Family history for CVD	Present	2.11 ± 0.86 (168)	0.048
	Absent	1.94 ± 0.73 (207)	

^*∗*^ *P* < 0.05 from  ^*∗∗*^. Control subjects have either normal fasting plasma glucose (<5.6 mmol/L), impaired FPG (5.6 to <7.0 mmol/L) or diabetic (≥7.0 mmol/L); HDL: high density lipoprotein; LDL: low density lipoprotein; TG: triglycerides; BMI: body mass index; HbA1c: hemoglobin A1c; CVD: cardiovascular disease. Except for diabetic status, all subjects were classified according to indicated parameters regardless of having diabetes or not.

**Table 3:** Correlation analysis matrix showing covariability between the study continuous variables.

	Age (years)	Systolic BP (mm Hg)	Diastolic BP (mm Hg)	FPG mmol/L	HbA1c (%)	TC mmol/L	TG mmol/L	HDL mmol/L	LDL mmol/L	BMI	DM years	TAS
Age (years)												
*r *	1											
*p *												
Systolic BP (mm Hg)												
*r *	0.267^*∗∗*^	1										
*p *	0.000											
Diastolic BP (mm Hg)												
*r *	0.111^*∗*^	0.582^*∗∗*^	1									
*p *	0.024	0.000										
FPG mmol/L												
*r *	0.143^*∗∗*^	0.127^*∗∗*^	0.106^*∗*^	1								
*p *	0.004	0.009	0.031									
HbA1c (%)												
*r *	0.204^*∗∗*^	0.234^*∗∗*^	0.151^*∗∗*^	0.776^*∗∗*^	1							
*p *	0.000	0.000	0.002	0.000								
TC mmol/L												
*r *	0.102^*∗*^	0.171^*∗∗*^	0.108^*∗*^	0.127^*∗∗*^	0.132^*∗∗*^	1						
*p *	0.038	0.000	0.027	0.009	0.008							
TG mmol/L												
*r *	0.057	0.150^*∗∗*^	0.114^*∗*^	0.178^*∗∗*^	0.220^*∗∗*^	0.348^*∗∗*^	1					
*p *	0.244	0.002	0.020	0.000	0.000	0.000						
HDL mmol/L												
*r *	0.015	0.051	0.033	−0.156^*∗∗*^	−0.172^*∗∗*^	0.260^*∗∗*^	−0.238^*∗∗*^	1				
*p *	0.763	0.301	0.503	0.001	0.000	0.000	0.000					
LDL mmol/L												
*r *	0.069	0.036	0.032	0.099^*∗*^	0.067	0.845^*∗∗*^	−0.013	0.194^*∗∗*^	1			
*p *	0.170	0.472	0.518	0.048	0.187	0.000	0.798	0.000				
BMI												
*r *	−0.030	0.092	0.146^*∗∗*^	0.101^*∗*^	0.090	−0.029	0.033	−0.012	−0.046	1		
*p *	0.539	0.060	0.003	0.040	0.070	0.560	0.505	0.809	0.362			
DM years												
*r *	0.285^*∗∗*^	0.133	0.059	0.312^*∗∗*^	0.408^*∗∗*^	−0.067	0.070	−0.016	−0.119	−0.245^*∗∗*^	1	
*p *	0.000	0.064	0.414	0.000	0.000	0.351	0.332	0.820	0.108	0.001		
TAS												
*r *	0.085	0.120^*∗*^	0.050	0.129^*∗*^	0.156^*∗∗*^	−0.008	−0.054	0.055	0.033	0.117^*∗*^	−0.140	1
*p *	0.100	0.019	0.334	0.012	0.002	0.876	0.296	0.286	0.527	0.022	0.055	

*r* = Pearson coefficient;  ^*∗∗*^ *P* < 0.01 level (two-tailed);  ^*∗*^ *P* < 0.05 level (two-tailed). FPG: fasting plasma glucose; HDL: high density lipoprotein; LDL: low density lipoprotein; TC: total cholesterol; TG: triglycerides; BMI: body mass index; HbA1c: hemoglobin A1c; DM: diabetes mellitus.

**Table 4:** Results of multivariate analysis for predictors of the TAS level.

Source		Adjusted TAS Mean ± SEM (*N*)	Sum of squares	*F*	*P *
Corrected model			22.415^a^	2.934	0.000

Intercept			4.130	7.027	0.008

Diabetic status	Control	2.020 ± 0.075 (195)	2.735	4.653	0.032
	Diabetic	1.896 ± 0.060 (197)			

Education	Illiterate	2.064 ± 0.101 (68)	5.089	2.887	0.036
	Less than high school	2.059 ± 0.060 (193)			
	High school	1.943 ± 0.116 (49)			
	Diploma and above	1.765 ± 0.108 (62)			

Family history for diabetes	Present	1.978 ± 0.061 (222)	0.039	0.067	0.796
	Absent	1.938 ± 0.075 (150)			

Family history for CVD	Present	2.032 ± 0.070 (165)	1.848	3.145	0.077
	Absent	1.884 ± 0.064 (207)			

Systolic BP			0.892	1.518	0.219

BMI			0.906	1.542	0.215

^a^ *R* Squared = 0.096 (adjusted *R* Squared = 0.063).

Analysis was performed using univariate general linear model (ANCOVA) as the potential predictors included categorical and continuous variables. TAS level was assessed as the dependent variable. Only variables that were significantly associated with TAS in the univariate analysis were included in the model. CVD: cardiovascular disease; BMI: body mass index; BP: blood pressure.
